# Synergistic use of anti-inflammatory ketorolac and gentamicin to target staphylococcal biofilms

**DOI:** 10.1186/s12967-024-04871-y

**Published:** 2024-01-25

**Authors:** Amita Sekar, Dmitry Gil, Peyton Tierney, Madeline McCanne, Vikram Daesety, Darina Trendafilova, Orhun K. Muratoglu, Ebru Oral

**Affiliations:** 1 Harris Orthopaedic Laboratory, Massachusetts General Hospital, Boston, USA; 2 Department of Orthopaedic Surgery, Harvard Medical School, Harvard University, Boston, USA

## Abstract

**Background:**

While antibiotics remain our primary tools against microbial infection, increasing antibiotic resistance (inherent and acquired) is a major detriment to their efficacy. A practical approach to maintaining or reversing the efficacy of antibiotics is the use of other commonly used therapeutics, which show synergistic antibacterial action with antibiotics. Here, we investigated the extent of antibacterial synergy between the antibiotic gentamicin and the anti-inflammatory ketorolac regarding the dynamics of biofilm growth, the rate of acquired resistance, and the possible mechanism of synergy.

**Methods:**

Control (ATCC 12600, ATCC 35984) and clinical strains (L1101, L1116) of *Staphylococcus aureus* and *Staphylococcus epidermidis* with varying antibiotic susceptibility profiles were used in this study to simulate implant-material associated low-risk and high-risk biofilms in vitro. The synergistic action of gentamicin sulfate (GS) and ketorolac tromethamine (KT), against planktonic staphylococcal strains were determined using the fractional inhibitory concentration measurement assay. *Nascent* (6 h) and *established* (24 h) biofilms were grown on 316L stainless steel plates and the synergistic biofilm eradication activity was determined and characterized using adherent bacteria count, minimum biofilm eradication concentration (MBEC) measurement for GS, visualization by live/dead imaging, scanning electron microscopy, gene expression of biofilm-associated genes, and bacterial membrane fluidity assessment.

**Results:**

Gentamicin-ketorolac (GS-KT) combination demonstrated synergistic antibacterial action against planktonic Staphylococci. Control and clinical strains showed distinct biofilm growth dynamics and an increase in biofilm maturity was shown to confer further resistance to gentamicin for both ‘low-risk’ and ‘high-risk’ biofilms. The addition of ketorolac enhanced the antibiofilm activity of gentamicin against acquired resistance in staphylococcal biofilms. Mechanistic studies revealed that the synergistic action of gentamicin–ketorolac interferes with biofilm morphology and subverts bacterial stress response altering bacterial physiology, membrane dynamics, and biofilm properties.

**Conclusion:**

The results of this study have a significant impact on the local administration of antibiotics and other therapeutic agents commonly used in the prevention and treatment of orthopaedic infections. Further, these results warrant the study of synergy for the concurrent or sequential administration of non-antibiotic drugs for antimicrobial effect.

**Supplementary Information:**

The online version contains supplementary material available at 10.1186/s12967-024-04871-y.

## Introduction

Orthopaedic infections are a heavy healthcare burden [1] because they are unpredictable and difficult to definitively diagnose, and their treatment is often lengthy and complex comprising multiple surgeries and medication regimens [2, 3]. Nevertheless, the recurrence rate is 20–35% and is associated with dire consequences such as arthrodesis [4, 5]. The 5-year survival rates of periprosthetic infection (PJI; ~ 70%) are like those of some cancers [6, 7]. Thus, PJI is a serious condition with high morbidity and mortality for patients that requires immediate attention. Most periprosthetic infections occur within 2 years of the index surgery. ‘Acute’ infections present with obvious signs such as swelling, or a sinus tract. In ‘chronic’ cases, the clinical manifestation is less clear where the patient may have some pain or discomfort without overt signs [8]. The current consensus is focused on using thresholds for systemic markers such as C-reactive protein and combining the results of blood tests such as that for leukocyte esterase to guide diagnosis and treatment decisions [9, 10]. The gold-standard approach is two-stage revision surgery which removes all implants followed by a period with intravenous (IV) antibiotics and an antibiotic-eluting bone cement spacer protecting the joint space in the meantime. When the infection is cleared (after about 4 months [11]), new devices are implanted. The cure rate of this approach is 54–77% [12].

Identification of bacteria, both in acute and chronic infections, is only possible by culturing intra-articular swabs. Treatment steps (medication and surgery) are often taken without knowing the details of the infecting bacteria [13] and there is a high probability of negative cultures (~ 20%) [8]. Despite these shortcomings of identification, the majority of positive cultures comprise gram-positive microorganisms *Staphylococcus aureus *(*SA*) and *Staphylococcus epidermidis* (*SE*) (60–70% [14]). In infected revisions, ~ 50% of Staphylococcal infections are methicillin-resistant [15]. Furthermore, coagulase-negative staphylococci (*S. epidermidis*) are known to persist due to their higher ability to form biofilms and are isolated from chronic infections more than other Staphylococcal species [16]. Thus, *Staphylococcus* spp. are the most relevant for the control of PJI and have varied characteristics.

Implant devices serve as substrates for biofilm attachment and colonization. The long-held view of the importance of initial bacterial attachment to medical device surfaces is based on work showing that these avascular surfaces can provide a safe harbor for bacteria to evolve into a state of biofilm that acquires resistance to soluble drugs [17–19]. Following adhesion, the biofilm is gradually established, with several subpopulations harboring varying phenotypic and genotypic signatures contributing significantly to the maturity and drug resistance [20]. Thus, it is believed that the rate of acquired resistance due to the formation of aggregates can be slowed down or eradicated by preventing the adhesion of bacteria to surfaces [21, 22].

The risk of treatment failure is a compound risk associated with the presence of staphylococcal infection, duration of the infection, host factors such as antecedent antibiotic exposure, immunocompetency, and the details of the treatment [23–25]. Current prevention strategy incorporating antibiotic therapy involves MRSA screening for methicillin-resistant *Staphylococcus aureus* (MRSA) in patients and peri-surgical systemic antibiotics [26–28]. The treatment generally involves systemic broad-coverage antibiotic therapy together with revision surgery to remove biofilm. Combination therapy utilizing beta-lactams/cell wall inhibiting antibiotics together with biofilm-targeting drugs such as rifampicin has proven the most effective current strategy in eradicating implant and tissue-associated biofilms [27]. Although inherent resistance to antibiotics is one of the major drivers of treatment outcomes, the implication of acquired resistance conferred by biofilm formation also warrants our attention. Antibiotics should be at an effective concentration at the site of infection for a significant period and they should be able to act on mature biofilms and persisters [29]. Effective clearance of infections becomes a growing challenge for antibiotics due to the inaccessibility of drug targets and emerging resistant bacterial subpopulations (persisters) due to the prolonged presence of drugs. This increases the burden on antibiotics and the immune system to access deep tissue spaces to target the bacteria [27]. Despite the challenges, antibiotics remain the mainstay of effective treatment, and increasing treatment success can be most likely obtained by targeting acquired resistance by bacteria and with strategies to use antibiotics more effectively. Altogether, the lack of such an efficient approach to eradicate biofilms contributes to a relatively high incidence of treatment failure [24].

Combination therapeutic strategies involving known antibiotics with other novel and repurposed non-antibiotic drugs are being explored to overcome the burden of bacterial resistance [30, 31]. We have been investigating the effect of common peri-surgical non-antibiotic therapeutics with antibiotics such as gentamicin which is one of the most used local antibiotics in orthopaedics due to its broad-spectrum activity [32]. Our findings were that the non-steroidal anti-inflammatory ketorolac tromethamine is synergistic with gentamicin [33]. Our focus is to devise local therapeutic delivery regimens based on a clinically relevant understanding of bacterial dynamics (growth and resistance acquisition) and with a realistic expectation of antibacterial activity [21]. This understanding can help us devise implants tailored to deliver the types and amounts of drugs specific to the expected ‘bacterial state’. It can also guide us in combining local delivery with other antibacterial tools at our disposal for maximum treatment success. This strategy incorporating drugs that are already in peri-surgical use (gentamicin and ketorolac tromethamine) is significant because it can potentially increase treatment success without additional risks to the patients. In this study, we are investigating the effect of gentamicin and ketorolac on planktonic bacteria as well as biofilms of laboratory and clinical strains of SA and SE in clinically relevant concentration ranges.

## Materials and methods

### Bacteria culture and maintenance

Control and clinical strains of *S. aureus* (ATCC 12600, L1101) and *S. epidermidis* (ATCC 35984, L1116) with varying antibiotic susceptibility profiles were used in this study (Additional file 1). All bacterial strains were thawed out from glycerol stocks stored at –80 °C and were cultured in sterile tryptic soy broth (TSB) or on tryptic soy agar plates (TSA) for a period of 18–24 h at 35 °C to achieve optimum growth prior to all experiments. The concentration of overnight bacterial suspension was spectrophotometrically determined at 600 nm and was enumerated using growth curves generated for each strain used in this study.

### Susceptibility testing of *Staphylococcal strains.*

The minimum inhibitory concentration [MIC] for GS and KT was determined to evaluate the susceptible and resistant nature of the Staphylococcal strains according to the Clinical and Laboratory Standards Institute (CLSI) protocol M07-A10 as described here. Briefly, the micro broth dilution assay was set up in a 96-well plate. GS and KT drug stocks were serially diluted in the wells using sterile Mueller Hinton Broth media (MHB) and 100 µL of diluted bacterial suspension (~ 10^5^ CFU) was added to each well containing a range of drug concentrations. Wells containing only bacteria and only blank media served as internal controls for the assay. The well plate was statically incubated at 35 °C for a period of 18–24 h. The minimum concentration which showed no turbidity was determined as the MIC of the drug.

The cumulative antibacterial effect of GS and KT on the Staphylococcal strains was determined by performing a checkerboard assay to measure the fractional inhibitory concentration as described previously [34]. Briefly, the overnight bacterial cultures were diluted to ~ 10^5^ CFU and exposed to different ratios of GS and KT combinations with MIC of each drug being the highest concentration combination tested. Following overnight incubation at 35 °C, the turbidity was visualized, and FIC indices were determined. The drug combination was considered synergistic if the ΣFIC < 1.0 [35].

### Evaluation of in-vitro biofilm dynamics

Staphylococcal bacterial suspension [10^5^ CFU/mL] in 1 mL of Luria–Bertani (LB) broth was inoculated on 316L Stainless Steel (SS) plates [10 × 3 × 1 mm] placed within 24-well plates. The SS plates were statically incubated for a period of 48 h at 35 °C. At each time point (6, 24, and 48 h), the spent media was removed, and the SS plates were washed thrice using sterile 1 × phosphate-buffered saline (PBS). The SS plates were transferred to 1.5 mL tubes and subjected to sonication for 40 min in 1 mL PBS to dislodge the adherent bacteria, The sonicate was then plated on tryptic soy agar plates and incubated for 18–24 h at 35 °C. The adherent bacteria count was determined the following day by the colony counting method.

### Determination of minimum biofilm eradication concentration (MBEC)

Staphylococcal biofilms were grown for a period of 6 h and 24 h on 316L SS coupons as previously described (Section “Evaluation of in-vitro biofilm dynamics”). The spent media was removed at each timepoint respectively and the SS plates were washed thrice with PBS to remove all non-adherent bacteria. The SS plates were then placed in a fresh 24-well plate containing GS and/or KT drug concentrations prepared in 10% LB-supplemented PBS solutions. The GS concentration range tested was (1–500 µg/mL) to determine the evolution of drug resistance in *nascent* (6 h) and *established* (24 h) biofilms. The KT concentrations tested were fixed at 0.5 mg/mL or 1 mg/mL (*nascent* biofilms) and 1 mg/mL or 3 mg/mL (*established* biofilms). Further to drug exposure for 24 h at 35 °C, the SS plates were gently rinsed thrice using PBS and were transferred to 1.5 mL tubes containing 1 mL PBS. The plates underwent sonication for 40 min and the adherent bacteria count was determined using the spread plate method. MBEC was determined as > 3log_10_ reduction in adherent bacteria count.

The MBEC of the GS-KT combination was visualized using the BacLight™ Live/Dead assay (Invitrogen, USA). Briefly, drug-treated *nascent* and *established* biofilms on SS plates (two per drug condition) were stained with SYTO 9/propidium iodide dye mixture according to the manufacturer’s protocol (1.5 µL of each dye per 1 mL of sterile deionized water). 200 µL of the diluted dye was dispensed on each of the stainless-steel plates for 30 min in the dark, followed by two washes with deionized water. The stained biofilms on SS plates were viewed using fluorescent microscopy (Nikon Ti2 Eclipse, Nikon Instruments Inc, USA, 400 × magnification). Ten fields were imaged per condition, with five randomly selected fields per plate. Propidium iodide-stained cells, and SYTO 9-stained cells were visualized using Texas Red and GFP filters, respectively. The resulting TIFF images were analyzed and quantified using the Biofilm Viability Checker plugin for Fiji (ImageJ™) image analysis software [36], which determined the proportion of live and dead bacteria.

### Molecular characterization of antibacterial activity of GS-KT

#### Scanning electron microscopy

Scanning electron microscopy was performed on the adherent bacteria on SS plates using the protocol described previously [37]. Briefly, the adherent bacteria were fixed using 2.5% glutaraldehyde in 0.1 M PBS for at least 48 h. The plates were then washed twice for 10 min with PBS. The adherent bacteria were then treated with 2% osmium tetroxide (OsO^4^) + 0.2% Ruthenium red (1:1) solution for a period of 1 h. The samples were washed twice thoroughly with distilled water for 10 min. Further to this, the samples were treated with 1% tannic acid for 30 min and then washed twice with distilled water for 10 min each. The prepared samples were imaged at 10–15 kV, high vacuum (Zeiss FESEM Ultra Plus).

#### Gene expression analysis

Adherent bacteria from *nascent* and *established* biofilms were exposed to indicated concentrations of GS and/or KT. The bacteria were harvested from SS plates (n = 10) for each condition. The sonicates were pooled and pelleted by centrifuging at 10,000 × g for 10 min. The pellet was then subjected to mechanical and enzymatic lysis and the total RNA was extracted using RNesay power biofilm kit protocol (Qiagen, Germany) for gram-positive bacteria. The RNA yield and quality were spectrophotometrically assessed using NanoDrop (Thermo Scientific, USA). RNA samples were converted to cDNA according to the iScript cDNA conversion kit protocol (Bio-Rad, USA). Real-time quantitative PCR was performed for *icaA*, *icaD*, *ebpS*, *vraR* genes for *S. aureus* and *icaA*, *icaD*, *atlE*, *vraR* for *S. epidermidis* using specific primers listed (Additional file 1). The Cq values were normalized to *S. aureus* and *S. epidermidis 16srRNA* expression, respectively. Comparative gene expression analysis was performed using the 2^^(−ΔΔCt)^ method [38]. No drug-treated *nascent* and *established* adherent bacterial gene expression served as control.

#### Membrane fluidity analysis

The membrane fluidity was investigated using the Laurdan assay [39]. Briefly, bacterial suspension [10^8^ CFU/mL] was centrifuged at 6000 × g, 5 min and the pellets were washed thrice with sterile PBS. The bacterial pellet was resuspended in 10 µM Laurdan reagent and incubated at dark for 10 min. The stained bacteria were pelleted and washed multiple times to remove excess stains. 100 µL of Laurdan-stained bacteria was then treated with indicated concentrations of GS and/or KT and the bound Laurdan fluorescence was read every 10 min for 40 min using a microplate reader. 50 mM benzyl alcohol treated bacteria served as the positive control. The fluorescence was read at excitation 350 nm and at two emission wavelengths 435 nm and 500 nm. The laurdan generalized polarization (GP) was calculated by measuring the fluorescence intensity (*i*) at 435 nm and 500 nm and using the following Eq.  [40].

$$Laurdan \,GP = \frac{i435nm - i500nm}{{i435nm + i500nm}}$$

### Statistical analysis

The gene expression studies were performed in triplicates and the non-parametric dataset was analyzed in R statistical software by performing a two-sided Wilcoxon Rank Sum Test using the function “wilcox.test”. The p-value was calculated and the lowest significant score of 0.1 was considered statistically significant.

## Results

### Gentamicin-ketorolac combination demonstrated synergistic antibacterial action against planktonic Staphylococci

The MIC values validated the gentamicin sensitivity level of all the strains (Table 1). The control SA demonstrated the lowest MIC for GS compared to that of the control SE and the clinical strains. KT demonstrated low growth inhibitory activity against the clinical strains compared to that against reference strains. The checkerboard assay was designed for each strain using the MIC values of the single drugs. GS-KT combination demonstrated ΣFIC < 1 for all staphylococcal strains indicating a synergistic/additive antimicrobial effect against planktonic bacteria which confirmed our previous findings for an expanded set of organisms and strains (Table 2) [33].

**Table 1:** MIC values for all the strains

Bacteria		Strains	MIC gentamicin (µg/mL)	MIC ketorolac (mg/mL)
*Staphylococcus aureus*	Control SA	ATCC 12600	1	8 ± 4
	Clinical SA	L1101	> 16	128 ± 2
*Staphylococcus epidermidis*	Control SE	ATCC 35984	> 16	8 ± 4
	Clinical SE	L1116	> 16	128 ± 2

**Table 2:** FIC indices for all the strains

Bacteria	Strains	Gentamicin-ketorolac [ΣFIC]
*Staphylococcus aureus*	ATCC 12600	0.8
	L1101	0.8
*Staphylococcus epidermidis*	ATCC 35984	0.9
	L1116	0.8

### Increasing biofilm maturity confers increased resistance to gentamicin for susceptible and resistant strains

**Fig. 1:**
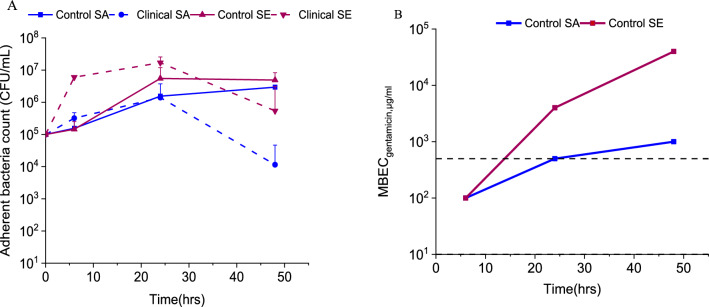
Staphylococcal biofilm growth dynamics and gentamicin resistance evolution over time. **A** Adherent bacteria count was determined to evaluate the biofilm dynamics of control (ATCC 12600, ATCC 35984) and clinical (L1101, L1116) Staphylococcal strains. **B** Gentamicin MBEC (minimum biofilm eradication concentration) evolution was determined over a period of 48 h for both control and clinical strains. The maximum concentration of gentamicin tested was capped at 500 µg/mL. Both 6 h and 24 h-grown biofilms of clinical strains demonstrated MBEC > 500 µg/mL

The differences in biofilm growth dynamics among staphylococcal species were characterized for both reference and clinical strains on 316L stainless steel plates. The control SA strain demonstrated a steady increase in adherent bacteria counts over 48 h. In contrast, the clinical SA strain showed a decline in viable (adherent) bacteria count after 24 h. The control SE strain, which possesses strong biofilm-forming capability, showed a sharp increase in viable adherent bacteria under 24 h and maintained high adherent viable bacteria counts even after 48 h. On the contrary, the clinical SE strain showed a drastic increase in adherent bacteria count in just under 6 h and a gradual decrease was observed after 24 h (Fig. 1A).

Biofilms show decreased susceptibility to antibiotics reducing the efficacy of treatments [41]. The role of biofilm dynamics in the significant reduction of susceptibility has not been well characterized with respect to medical device-associated infections. The MBEC_gentamicin_ (> 3log_10_ reduction) for a 6 h-grown biofilm of the reference SA strain was 100 $\pm$ 20 µg/mL, which subsequently increased to > 500 µg/mL at 48 h of growth. This result showed that increased biofilm maturity conferred increased drug resistance to an otherwise susceptible strain. The biofilm of the reference SE strain grown for 6 h demonstrated an MBEC of 100 $\pm$ 20 µg/mL, which drastically increased to > 500 µg/mL for 24 h and 48 h biofilms. On the other hand, biofilms of the clinical staphylococcal strains grown for 6, 24, and 48 h all exhibited MBEC > 500 µg/mL. The data showed the compounded effect of inherent and acquired resistance for these strains (Fig. 1B). Based on these data, we designated 500 μg/mL of gentamicin as a clinically feasible maximum concentration of interest. For the following experiments evaluating synergy, we designated biofilms grown for 6 h as ‘*nascent*’ and those grown for 24 h as ‘*established*’ based on the evolution of resistance against gentamicin.

### Ketorolac enhances the activity of gentamicin against acquired resistance

**Fig. 2:**
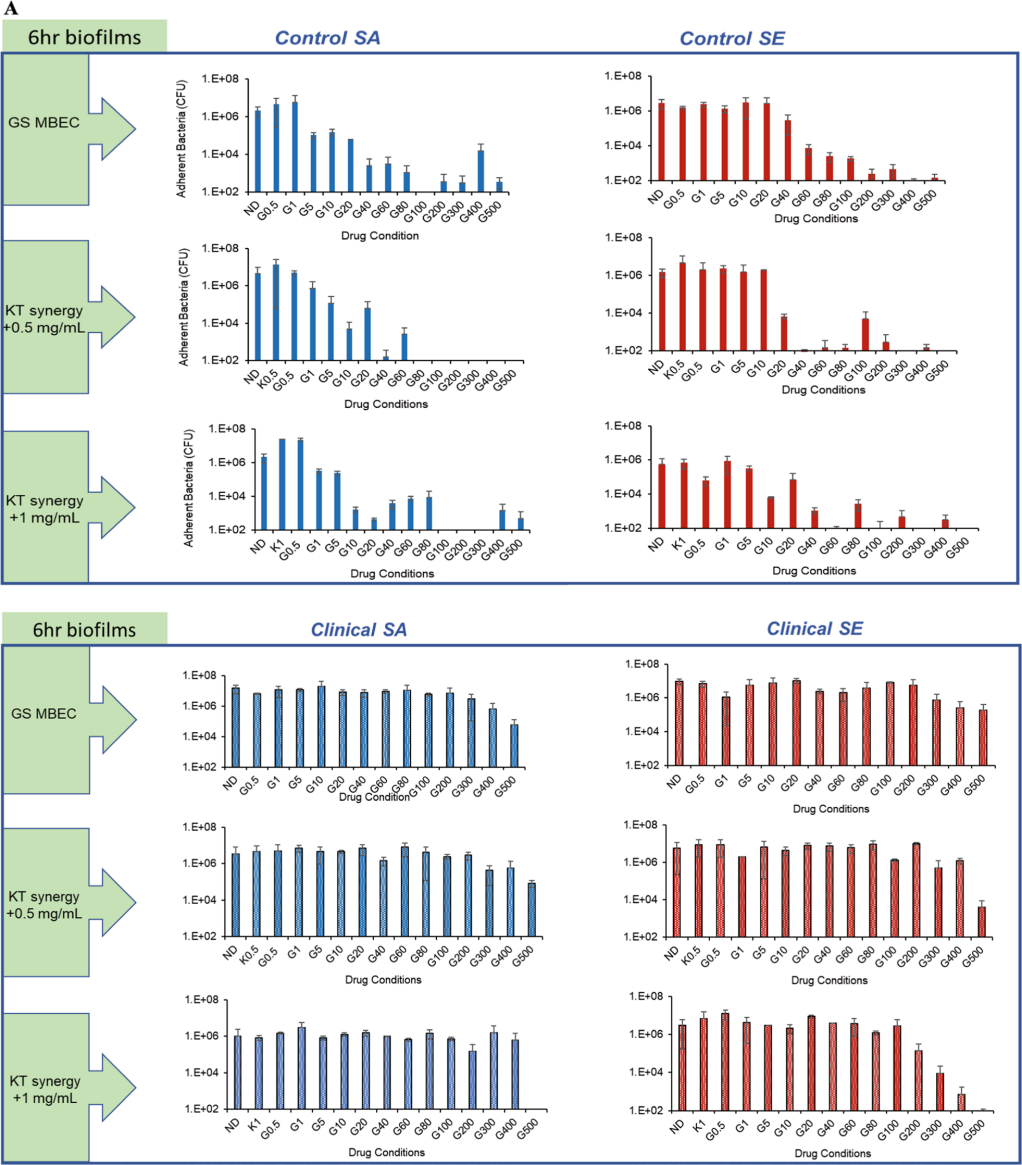

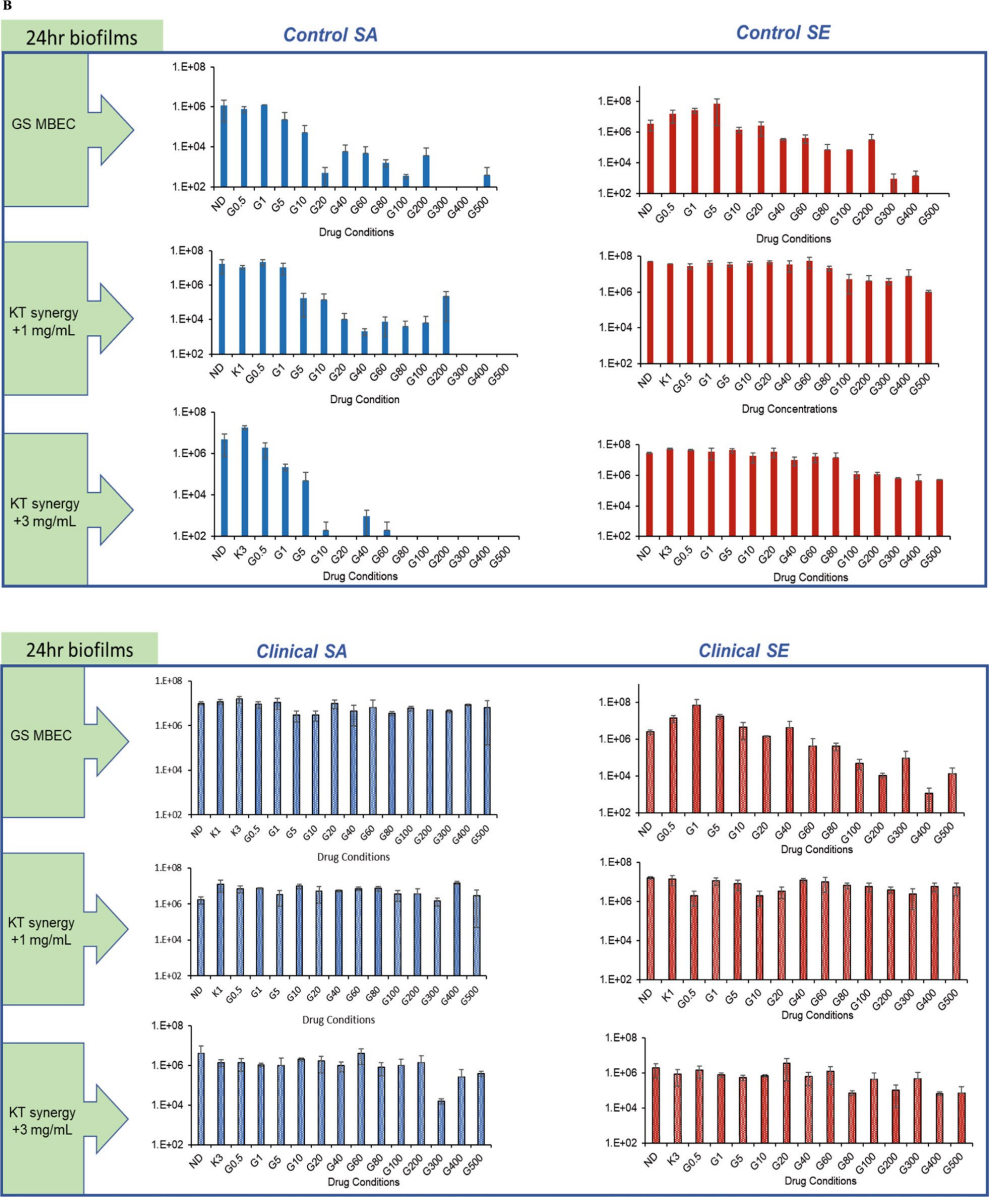

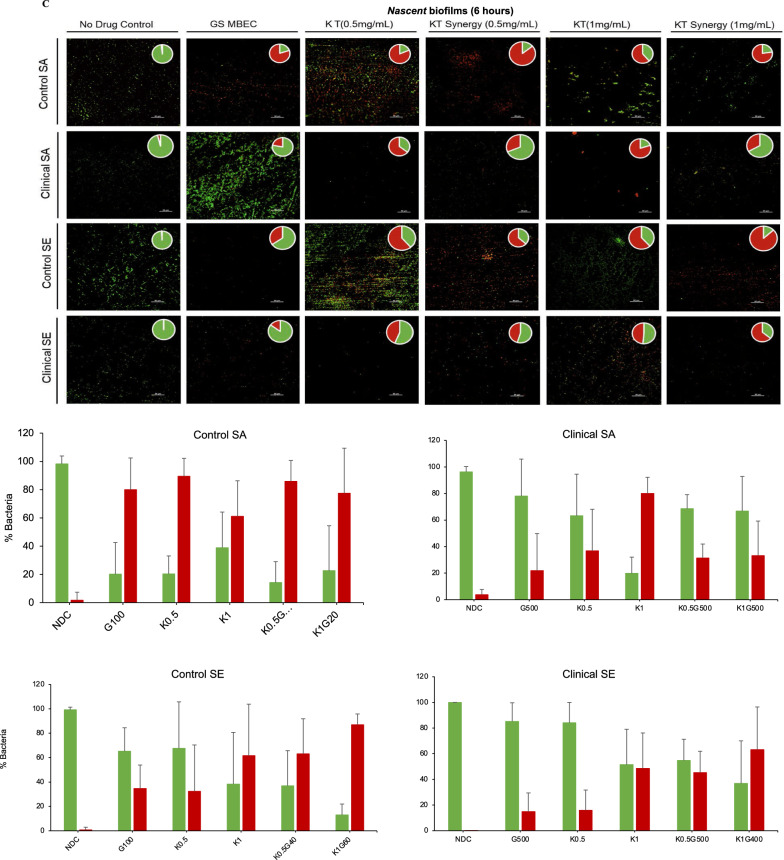

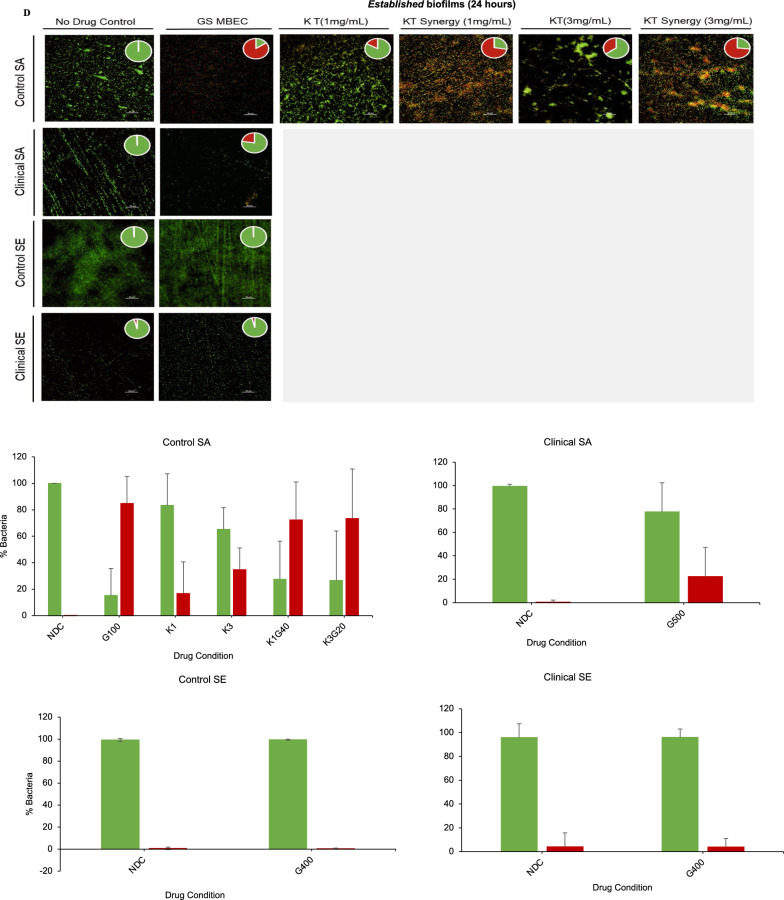
Addition of ketorolac enhances the activity of gentamicin against biofilm maturity-associated resistance in staphylococcal biofilms **A **Effect of KT synergy (0.5 mg/mL and 1 mg/mL) on *nascent* control and clinical staphylococcal biofilms. **B** Effect of KT synergy (1 mg/mL and 3 mg/mL) on *established* control and clinical staphylococcal biofilms. **C** Effect of KT synergy on *nascent* and **D** *established* biofilms observed using Live/Dead staining (scale bar = 50 µm). The live/dead adherent bacteria percentage was quantified and represented as a bar plot. The error bars represent the standard deviation (n = 10)

We investigated the effect of the use of KT to determine the range of concentrations for gentamicin that are efficacious against the strains with different susceptibility, including those with increased inherent or acquired resistance. The KT concentrations used were below the MIC of KT (Table 1) and in the clinically feasible range for pain management by KT administration [42, 43]. The addition of 0.5 and 1 mg/ml of ketorolac significantly decreased the MBEC for the *nascent* biofilms of all tested strains (Fig. 2A). For the bacteria in the *established* biofilms of the control SA strain with increased acquired gentamicin resistance, there was a > 3log_10_ reduction with the addition of 1 and 3 mg/mL KT at gentamicin concentrations of 40 and 20 µg/mL, respectively. The bacteria in the biofilms of the inherently resistant clinical strains were not eradicated at gentamicin concentrations below the threshold concentration with the addition of indicated KT (Fig. 2B). The MBEC values for gentamicin against the *nascent* and *established* biofilms with and without the addition of KT are summarized in Table 3 and were used for all subsequent experiments evaluating the extent and mechanism of the synergy.

**Table 3:** GS MBEC values for *nascent* and *established* biofilms with and without the addition of indicated concentrations of KT

Biofilm maturity	Bacteria	Strain	MBEC (GS, µg/mL)	+ 0.5 mg/mL KT	+ 1 mg/mL KT
*Nascent* (6 h)	*S. aureus*	ATCC 12600	100	60	20
		L1101	> 500	500	500
	*S. epidermidis*	ATCC 35984	200	40	60
		L1116	> 500	500	400
**Biofilm maturity**	**Bacteria**	**Strain**	**MBEC** **(GS, µg/mL)**	** + 1 mg/mL KT**	** + 3 mg/mL KT**
*Established* (24 h)	*S. aureus*	ATCC 12600	100	40	20
		L1101	> 500	−	−
	*S. epidermidis*	ATCC 35984	400	−	−
		L1116	400	−	−

The synergistic antibiofilm effect was further confirmed in real-time by quantifying the viability of the biofilm using the Live/Dead assay. The *nascent* biofilms of the control SA, SE, and clinical SE strains demonstrated viability loss with the addition of KT, which was either comparable or more than the observed viability loss for biofilms exposed to GS treatment alone at MBEC. For the inherently resistant clinical SA strain, both GS and synergistic GS-KT combination treatment did not show a real-time impact on bacterial viability. The treatment of the clinical SA biofilm with 1 mg/mL of KT alone without GS demonstrated viability loss (Fig. 2C), suggesting that the variability of the bacterial eradication capability of KT needs further study for multiple strains including multi-drug resistant ones. The synergistic action against biofilm-associated acquired resistance was also determined for *established* biofilms of the reference SA strain. The addition of 1 and 3 mg/mL KT in combination with 40 µg/mL and 20 µg/mL of GS, respectively, reduced bacterial viability which was comparable to the biofilms treated with GS at MBEC (100 µg/mL) (Fig. 2D), in line with the plate culture. On the other hand, the biofilms of the inherently resistant strains (clinical SA, control SE, and clinical SE) showed no significant biofilm viability loss in the presence of GS.

### The addition of KT interferes with extracellular matrix formation and biofilm morphology

**Fig. 3:**
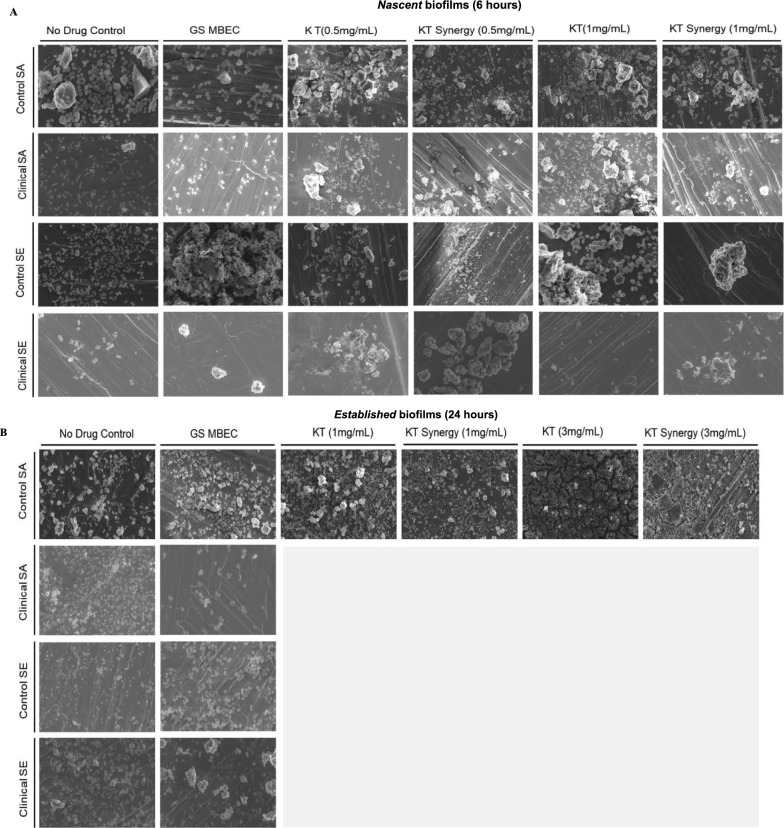
Visualization of effect of synergy on biofilm morphology Scanning electron micrographs of (**A**) *nascent* and (**B**) *established* biofilms exposed to GS MBEC and KT synergy combinations (scale bar = 2 µm, magnification = 2.5KX)

Scanning electron microscopy was performed to visually examine *nascent* and *established* biofilms when exposed to GS and GS together with KT. The images were qualitatively analyzed for the differences in the extracellular matrix density and morphology, bacterial aggregation pattern, density, and the integrity of the cell wall. A series of images were taken after exposing the biofilms grown on SS plates to the effective concentration of GS, synergistic combination of GS/KT and control concentration of KT indicated in Table 3. The untreated biofilms exhibited more intact bacterial aggregates attached to the SS plate. The connective ECM structures were more abundant in the biofilms of the control strains compared to those of the clinical strains (Fig. 3A).

*Nascent biofilms of control SA* treated with K1 synergy concentration showed sparse bacterial density and an overall reduction of bacteria and bacterial aggregation. These observations were similar to those in the biofilms exposed to GS only at MBEC. Morphologically, the K1 synergy combination resulted in more fractured extracellular matrix (ECM) structures compared to other conditions. Additionally, the bacterial spheres attached to the ECM structures were reduced in size compared to the bacteria seen in the GS MBEC-treated and no drug-treated conditions.

For the *nascent biofilms of clinical SA*, the K1 synergy condition showed a small deposition of ECM with little to no attached cells and the remnants of deflated spheres of individual bacteria. These features were similar to those of the sparsely distributed bacteria exposed to GS only at MBEC (Fig. 3A). Thus, the imaging confirmed that exposing *nascent* biofilms to synergistic combinations of GS/KT (where GS concentration < MBEC) resulted in similar biofilm features to when they were exposed to GS only at MBEC.

In *nascent biofilms of control SE*, there was little ECM production (Fig. 3A). Control SE biofilm exposed to K1 synergy concentration demonstrated sporadic ECM structures studded with a few viable bacteria. This was in stark contrast to the biofilms exposed to GS only at MBEC, which showed dense ECM with copious embedded bacteria and bacteria agglomerates.

*The nascent biofilms of the clinical SE strain* exposed to the K1 synergy concentration exhibited sparsely distributed matrix debris and deflated bacteria (presumably non-viable). These features were similar to those of biofilms exposed to GS only at MBEC (Fig. 3A).

*The established biofilms of control SA* showed greater surface coverage of the SS plate compared to that of the *nascent* biofilms, with intact bacterial populations and connected ECM structures (Fig. 3B). Although biofilms treated with GS only at MBEC appeared thinly dispersed with reduced viability, the biofilms exposed to the K3 synergy concentration showed larger areas of fragmented ECM structures in addition to the reduced viability of bacteria.

*The established biofilms of clinical SA* showed deflated, dispersed bacteria with little ECM when exposed to GS only at MBEC. *The established biofilm of the control SE* showed denser bacterial agglomerates with little ECM and *the established biofilm of the clinical SE* showed similar features to that of the clinical SA. There were no synergistic combinations of GS/KT for GS concentrations less than 500 μg/mL for *established* biofilms of these three strains. The most visible effect of the presence of KT was the increased production of ECM. The synergistic use of GS/KT affected the bacterial viability and decreased the structural integrity of the ECM for the biofilms susceptible to it. These results also suggested an influence of ketorolac on the balance between resistance acquisition and other processes, such as matrix production.

### GS-KT synergy subverts bacterial stress response and alters biofilm properties

**Fig. 4:**
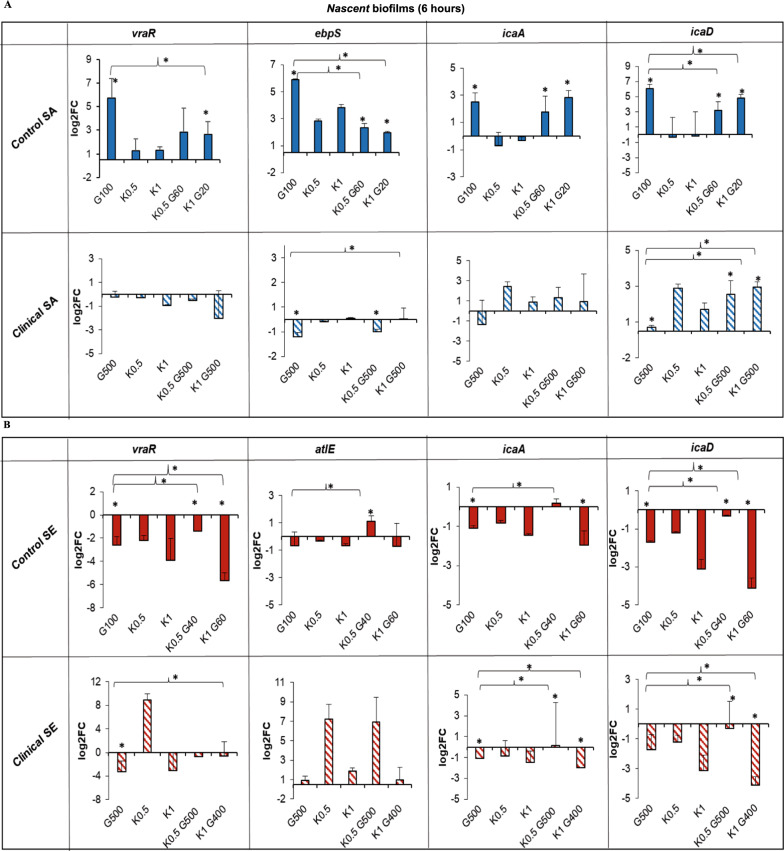

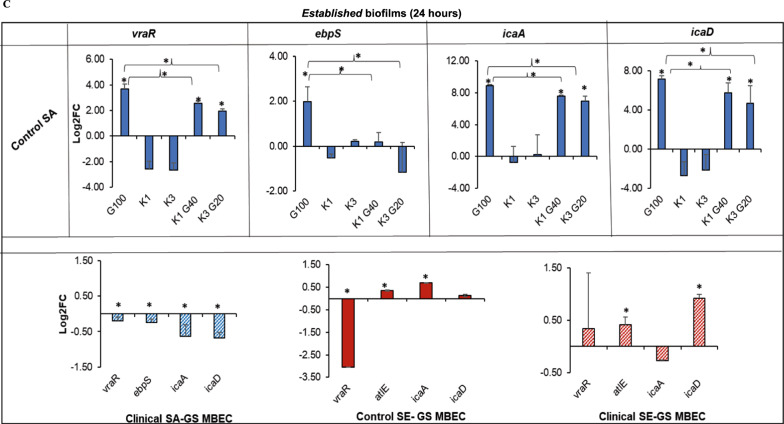
Characterizing effect of synergy on vital bacterial processes and responses Gene expression analysis of *vraR*, *icaA*, *icaD*, *ebpS*, and *atlE* genes in *nascent* (**A**, **B**), and *established* biofilms (**C**) of control and clinical staphylococci. Error bars represent the standard deviation of 3 replicates. (Wilcoxon rank sum test; *indicates p-value of 0.1)

To elucidate the changes in bacterial stress responses triggered by exposure to GS at MBEC and at synergistic combinations with KT, gene expression studies were performed (Fig. 4A–C). The effect of exposing the bacteria to MBEC on the expression of biofilm-associated *icaA, icaD* genes, bacterial adhesion-associated *ebpS, and atlE* genes (for *S. aureus* and *S. epidermidis,* respectively) and peptidoglycan biosynthesis-associated *vraR* gene were studied.

*In nascent biofilms of control SA*, exposure to GS at MBEC significantly increased the *vraR*, *ebpS*, *icaA,* and *icaD* expression when compared to that with no drug exposure. Concurrently, exposure to the K0.5 synergy and K1 synergy concentrations also demonstrated upregulation for all the genes to a lesser degree when compared to the expression of biofilms exposed to GS at MBEC. The data indicated similarities in the stress responses triggered by GS at MBEC and the synergistic concentrations with KT, which agreed with the qualitative visual cues obtained using SEM, such as the size and density of bacterial aggregates. The presence of KT alone did not show a difference in gene expression except for that of eb*pS*, which revealed that adhesion was upregulated (> 1.5-fold) for all conditions (Fig. 4A).

*In nascent biofilms of clinical SA*, the expression of the four studied genes was not changed when the bacteria were exposed to GS at MBEC, supporting the response expected from this resistant strain in the presence of gentamicin. However, in the presence of KT, and its synergistic combinations with GS, *icaD* expression was significantly upregulated (~ threefold) as well as *vraR* (> twofold), and *icaA* (onefold) and no change was observed for *ebpS* (Fig. 4A). This data supported the real-time viability loss observed in the presence of KT for *nascent* clinical SA biofilms at high GS concentrations (Fig. 2C). The increase of icaA and icaD gene expression supported the SEM observations for the clinical SA, which showed more aggregation of bacteria. The size of the bacteria was small, which may be attributed to increased drug resistance and higher vraR expression [44].

*In nascent biofilms of control SE*, bacteria with exposure to GS at MBEC demonstrated significant down regulation of *vraR* (> twofold), *icaA* (onefold), and *icaD* (> twofold) genes. The exposure to the K1 synergy combination (KT 1 mg/mL + GS 60 µg/mL) triggered a further reduction in gene expression levels for *vraR* (> fivefold), *icaA* (twofold), and *icaD* (fourfold), revealing a notable enhancement of antibiofilm properties of GS with the addition of KT (Fig. 4B). The gene expression data correlated with observations from SEM images and viability data exhibiting significantly diminished colony size, less bacterial aggregates, reduced slime production, and viability loss, respectively, for *nascent* control SE biofilms (Fig. 2C and 3A). On the contrary, adhesion-associated *atlE* showed a slight but significant increase when compared to GS at MBEC and no drug control. The overall increased expression of *atlE*, which is the most prominent SE adhesin, could be attributed to the increased adhesion capability of SE strains [45].

*Nascent biofilms of clinical SE* exhibited a significant reduction in *icaA, icaD,* and *vraR* in the presence of GS at MBEC compared to those without drug exposure. The presence of KT in the synergistic combinations, specifically K1 synergy, increased the deregulation of *icaA* and *icaD* genes compared to biofilms exposed to GS at MBEC. The synergistic combinations did not show a difference in the regulation of the *vraR* gene expression compared to that of the biofilms exposed to GS at MBEC. This indicated that the addition of KT suppressed the matrix production triggered by GS. The expression of the adhesion-related atlE gene was upregulated by exposure to the K0.5 synergy combination compared to exposure to GS at MBEC; however, the change was not statistically significant (Fig. 4B). The data agreed with the visual observations of pronounced antibiofilm activity exerted by the synergistic combination of GS and KT (Figs. 2C and 3A).

For the *established biofilms of the control SA strain*, exposure to GS at MBEC and to K1 and K3 synergy combinations significantly upregulated the expression of *vraR* (> twofold), *icaA* (eightfold), and *icaD* (> fivefold) when compared to those without drug exposure (Fig. 4C). The bacterial molecular responses triggered by the exposure to both GS at MBEC and its synergistic combinations with KT, as determined by gene expression profiles, were consistently similar in *established* biofilms. This behavior was markedly different from that of *nascent* biofilms, where the addition of KT significantly reduced the gene expression levels of *ebpS* compared to biofilms exposed to GS at MBEC, which indicated compromised adhesion in biofilms exposed to KT synergy combinations. The data obtained aligned with the viability assay and SEM observations, indicating GS and KT synergistically work to impact biofilm adhesion, matrix formation, and biofilm-associated resistance to antibiotics (Figs. 2D and 3B).

Due to the absence of effective synergistic combinations against the *established* biofilms of *clinical SA, control SE, and clinical SE strains,* the gene expression analysis was performed only for biofilms exposed to GS at MBEC. For the clinical SA, there were low levels of expression for all four genes but there were significant differences when compared to biofilms with no drug exposure. In contrast, *vraR* expression in *control SE biofilms* was significantly downregulated (> 2.5-fold) in the presence of GS at MBEC along with a < onefold increase for *atlE* and *icaA* genes. In the *clinical SE strain,* GS exposure at MBEC triggered a significant but small upregulation effect for *atlE* (0.5-fold) and *icaD* (onefold) expression (Fig. 4C). The data validated the observations from the live/dead viability assay and SEM indicating an overall subdued response of these strains against GS.

### The addition of KT triggers staphylococcal membrane rigidification

**Fig. 5:**
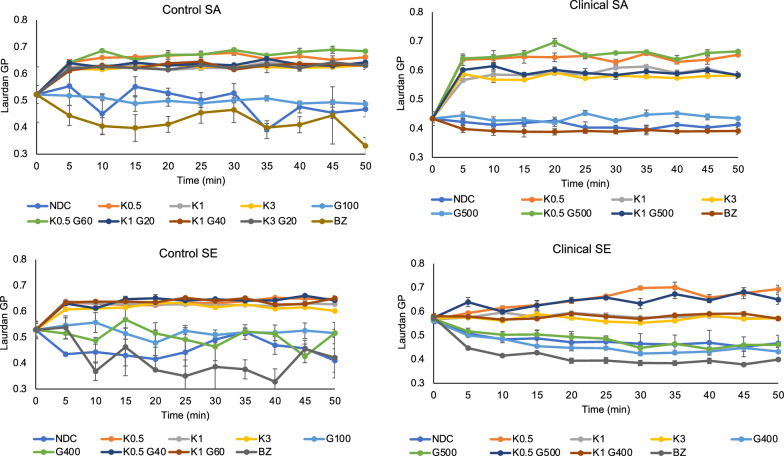
Characterizing effect of synergy on bacterial membrane dynamics Membrane fluidity (Laurdan GP) was determined for the control and clinical staphylococcal strains exposed to GS MBEC and KT synergy combinations. The line graphs denote Laurdan GP values plotted over a period of 40 min. Error bars represent the standard deviation (n = 3)

The antibacterial investigation of exposure to KT, in addition to GS and the demonstration of efficacy, especially in hindering acquired resistance of *nascent* biofilms, suggests an alternative mechanism of action for KT. Antibiotics are known to impact bacterial membrane fluidity during their course of action [46, 47]. The antibacterial activity greatly depends upon the capability of antibiotics to insert themselves into the bacterial membrane or access the inner physiological system through specific channels across the membrane [48, 49]. To understand the mechanism of action of GS at MBEC and its synergistic combinations of KT on the bacterial membranes of staphylococcal strains, bacterial membrane fluidity was determined using Laurdan fluorescence [39]. The addition of KT dramatically increased the Laurdan GP values (> 0.5), exhibiting membrane rigidifying properties regardless of the presence of GS. Interestingly, the GS at MBEC-exposed bacteria showed average Laurdan GP values close to < 0.5, indicating little to no fluidizing properties of GS within the time frame of the experiment (40 min) when compared to no-drug control. Bacteria exposed to benzyl alcohol as a membrane fluidizer served as the positive control (Laurdan GP values < 0.4) (Fig. 5). The data validated the nature of killing kinetics of gentamicin which is known to be partially concentration-dependent and that higher concentrations of gentamicin do not aid in additional eradication due to adaptive resistance mechanisms [50, 51]. A novel membrane-rigidifying molecular action of KT was revealed which seems to facilitate the synergistic antibacterial action with GS.

## Discussion

The elucidation of the translational value of antibiotic-analgesic synergistic use in treating infections holds significant promise for improving patient and overall healthcare outcomes [52]. The outcomes of PJI are often complicated by virulence, biofilm formation, innate antibiotic resistance, and persistence [53]. Understanding the risk and severity of the infection is paramount for designing the best therapeutic approaches that would result in the effective eradication of the infection without the development of resistance or persister populations. Current approaches focus on delivering a pre-determined cocktail of antibiotics for PJI patients without clarity about the status of infecting organisms [53, 54]. This 'one size fits all’ therapeutic strategy has not hindered the increasing incidence in infections leading to significant morbidity and mortality [55, 56]. Postoperative administration of antibiotics is used to prevent PJI [57] and of local anesthetics/analgesics to address peri-surgical pain [58]. Combined local use of antibiotics and analgesics to target infections could aid in reducing the inappropriate use of antibiotics and hindering the development of antibiotic resistance [54, 59]. Previously, we showed that several commonly used analgesics and NSAIDs yield pronounced synergistic/additive antibacterial effects against PJI-causing planktonic Staphylococci when used in combination with antibiotics in vitro [33]. In this study, we are exploring and optimizing the application of the NSAID ketorolac in combination with the antibiotic gentamicin, to synergistically enhance its efficacy, allowing for better control of staphylococcal biofilms.

Synergy has been investigated and reported largely for multiple antibiotics [60]. In the face of the emergence of multi-drug resistant organisms, antibiotic synergy with non-antibiotics has also gained interest [31, 53]. Most of the work has focused on the synergy between antibiotics and non-conventional antimicrobial strategies such as the use of bacteriophages, antimicrobial peptides, and small molecules, inhibiting vital bacterial processes [47, 61–64]. Recently, there has also been considerable interest in repurposing non-antibiotic drugs for the purpose of infection prevention and management [65]. In a post-operative care setting, local application of antibiotics and non-antibiotic drugs are common to prevent the onset of PJI and pain management. However, little is known about the repurposing of nonantibiotic drugs (locally) to enhance the activity of existing antibiotics and no combinations have been investigated specifically for indications related to periprosthetic (bone) infections. Here we explored the combined antibacterial activity of two drugs used in local applications for total joint arthroplasty to explore the enhancement of antibacterial activity against *nascent* (6 h) and mature (24 h) staphylococcal biofilms.

Different strains harboring varying resistance profiles were used to simulate a ‘low-risk’ and ‘high-risk’ infection and to understand the range of applicability of the proposed dual use of gentamicin and ketorolac against eradicating biofilms of varying risk and severity. There were strain-specific differences in the biofilm formation rate and viable bacterial recovery at each time point for in-vitro-grown biofilms. When the *nascent* and *established* biofilms were subjected to a range of gentamicin concentrations, an increase in gentamicin resistance commensurate with increasing biofilm maturity was observed. Thus, using two strains with differing inherent resistance in combination with their differing time-dependent acquisition of resistance due to biofilm formation gave us a wide range of ‘bacterial states’ against which to test synergy.

The primary goal of this study was to determine whether a non-antibiotic compound can be used to work in tandem with the antibiotic to overcome biofilm-associated acquired resistance. To this end, low concentrations of ketorolac close to clinical dosing guidelines were used to determine the synergistic action with gentamicin against biofilms. The GS-KT combinations demonstrated a pronounced effect against biofilm-associated acquired resistance in staphylococcal strains. The synergistic application evidently resulted in significant morphological and physiological changes that impacted bacterial viability, cell size, cell wall structures and biofilm matrix production (Figs. 2–3). The findings from this study reveal that the synergistic use of ketorolac with gentamicin, for example, that is eluted from antibiotic-eluting bone cement, could be evaluated as a prophylactic measure to reduce the antibiotic load and increase treatment efficacy.

The critical factors for staphylococcal biofilm formation are the presence of abiotic surfaces and damaged peri-implant tissues in vivo [66]. The bacterial colonization process is associated with the expression of several adhesins, slime production, and stress-induced pathways [45]. These aid the bacteria to effectively attach to surfaces, evade host response to infection, and resist antibiotic treatments. Understanding the regulation of these molecular events, specifically in response to the presence of antibacterial agents, is crucial to determining the antimicrobial dynamics. *S. aureus* is known to elicit cell wall-associated gene expression in the presence of antimicrobial agents, a well-documented phenomenon [67–69]. These cell wall responses are directly linked to the emergence of small colony variants and antibiotic resistance [44, 70, 71]. Within the scope of our study, the bacterial response to restore the cell wall integrity (indicated by *vraR* expression) was overall subdued in the presence of GS-KT, which was validated by the compromised cell morphology and diminished cell size observed under SEM. The lack of such a crucial bacterial response to maintain the cell wall components in the presence of GS-KT could be advantageous for gentamicin’s easy access into bacterial cells. Vital pathogenic processes such as adhesion and biofilm formation were also investigated. EbpS and AtlE, established adhesin markers for SA and SE biofilms, respectively, have been identified to initiate bacterial interactions with host factors and surfaces to promote attachment [72–74]. In our study, GS treatment markedly augmented adhesin expression in *nascent* biofilms of control SA, an effect mitigated by the addition of KT. This strongly suggests a potential increase in bacterial attachment facilitated by commonly used gentamicin in a ‘low-risk’ infection, while the addition of KT attenuated this process. However, in clinical SA biofilms, the expression remained largely unaltered, correlating with its expression being tied to the presence of soluble elastins [73]. In SE biofilms, irrespective of maturity, adhesin expression did not show an increased expression, which correlated with it being important later in the biofilm cycle [45].

Biofilm formation, which is mediated by the *icaADBC* locus [75], was found to be differentially regulated in a strain-dependent manner by GS-KT synergy. Among the two strains, SE is known to be the high slime producer clinically [66, 76] and it was interesting to observe the drastic deregulation of *icaA* and *icaD* gene expression triggered by GS and GS-KT combinations. This data strongly emphasizes the potential of the synergistic application against biofilm formation, as this is the major virulence factor for pathogenesis. Conversely, when exposed to GS at MBEC and GS-KT combinations, a notable upregulation in the biofilm-associated gene expression was observed in both *nascent* and mature SA biofilms. These findings aligned with other studies reporting an increase in staphylococcal biomass in the presence of gentamicin [77]. The gene expression overall complemented the SEM observations to a certain extent, but additional studies on other genes mediated by the *ica* locus are needed to reveal if the synthesized slime translocation and deacetylation pathways are affected by drug treatment [78]. The nutrient-dependent uptake and activity of gentamicin in the presence of ketorolac should also be evaluated to further understand the synergy dynamics. These preliminary findings aid in the understanding of the mechanism of action of GS-KT synergy. For all the ‘high-risk’ mature biofilms where the GS-KT combinations were ineffective, alternative analgesic and anti-inflammatory combinations with known antibiotics are being explored.

The gene expression data regarding the cell wall biosynthesis response of bacteria in the presence of GS at MBEC and GS-KT revealed that the addition of ketorolac significantly subdues or negatively regulates the bacterial pathways to restore membrane integrity. The bacterial lipid bilayers consist of rigid and fluid domains that determine the bacterial membrane fluidity, and they adapt quickly to changing environments [79]. Laurdan reagent is capable of intercalating to the bacterial lipid bilayer and can assess the membrane fluidity based on the number of water molecules around the Laurdan molecule [39, 80]. This attribute makes it an excellent probe to understand the mechanism of antimicrobial action. Aminoglycosides are internalized due to the presence of proton motive force (PMF) and the influx of gentamicin is known to cause increased membrane fluidity [81]. Biofilm formation decreases the overall metabolic activity and growth, leading to a diminished PMF imparting resistance to these antibiotics [82]. Biofilm maturity is a factor in increased resistance to antibiotics, and it is highly likely due to a diminished PMF [83]. Recent reports have revealed that non-antibiotic agents can induce PMF-independent uptake of aminoglycosides by altering membrane potential [81]. In our study, the addition of KT was primarily found to rigidify bacterial membranes in a concentration-independent manner. The data was similar to that of the antibiotic daptomycin, which decreases membrane potential (dose-dependently) by clustering lipid domains due to its insertion into the bacterial membrane [84, 85]. These observations strongly suggest that the membrane destabilization caused by KT could improve the uptake of gentamicin into the biofilms in a PMF-independent manner and thereby target the biofilm maturity associated-gentamicin resistance.

Our results firstly showed possible differences in PJI biofilms in vitro and the possible application of stratifying the risk of infections while modeling clinically relevant situations. The adaptation of such a risk stratification strategy can enable the design of drug delivery vehicles and profiles according to the inherent resistance profile, biofilm composition, and molecular status of bacteria to improve treatment outcomes. Our study also revealed the potential of the synergistic use of an analgesic in combination with an antibiotic as a novel prophylactic approach to prevent PJI. The design of functional implants for the local sustained delivery of antibiotics, as well as the delivery of synergistic combinations of drugs, can be possible based on our work on polymeric drug delivery devices [86–89].

Our study is limited to in-vitro culture: there is very little known about the progression of in-vivo infections, especially in the early stages where prevention and treatment may be more probable. The diagnosis of infections in vivo is characterized by clinical symptoms and there are gaps in our knowledge about the characterization of the bacterial state and its changes over time. Thus, further study to understand the relationship between bacterial populations grown in vivo and in vitro is required. Similarly, the efficacy of antibiotic drugs can be affected by other factors in the in-vivo environment such as the binding of proteins and the clearance rate from the local environment. Finally, the host immune system has an integral role in determining the efficacy of any administered antibiotics as the major force in detecting, disarming, and clearing infections, which were not modeled in our study.

## Conclusion

In summary, the synergistic effect of post-operative anti-inflammatory drugs enhancing the antibacterial activity of common prophylactic antibiotic gentamicin was shown for the first time. Our findings also emphasize the importance of infection risk assessment to be used as a tool to design better prophylactic and therapeutic approaches.

### Supplementary Information

**Additional file1: Table S1.** Susceptibility profile of strains used in the study. **Table S2.** Primer sequences for gene expression study.

## Data Availability

The data supporting the conclusions of this article are included within the article. More information on materials and methods are provided in Additional file 1.
