# Supplementary material for: Synergistic use of anti-inflammatory ketorolac and gentamicin to target staphylococcal biofilms
Source: J Transl Med. 2024 Jan 25;22:102. doi: 10.1186/s12967-024-04871-y (PMC10809490; doi:10.1186/s12967-024-04871-y)
Supplement: Supplementary file 1 — Additional file1: Table S1. Susceptibility profile of strains used in the study. Table S2. Primer sequences for gene expression study. [file 12967_2024_4871_MOESM1_ESM.pdf]

Table 1: Susceptibility profile of strains used in the study.

Bacteria	Strain	Methicillin	Gentamicin	Vancomycin
Staphylococcus aureus	ATCC 12600 (control)	Susceptible	Susceptible	Susceptible
	L1101 (clinical)	Resistant	Resistant	Intermediate
Staphylococcus epidermidis	ATCC 35984 (control)	Resistant	Resistant	Susceptible
	L1116 (clinical)	Resistant	Resistant	Intermediate

Table 2: Primer sequences for gene expression study

Staphylococcus aureus	
vraR	FP 5’-AACTCTGCGCGCTTTTTCAT-3’
	RP 5’-ATATCGCCGATGCAGTTCGT-3’
icaA	FP 5’-TTGTCGACGTTGGCTACTGG-3’
	RP 5’-GCGTTGCTTCCAAAGACCTC-3’
icaD	FP 5’-CGCTATATCGTGTGTCTTTTGGA-3’
	RP 5’-TCGCGAAAATGCCCATAGTT-3’
ebpS	FP 5’-TACTTTGGCCATGCCACCTT-3’
	RP 5’-TGCTTCTGCCGCTTCAAAAC-3’
16srRNA	FP 5’-AGACCAGAAAGTCGCCTTCG-3’
	RP 5’-TCAACCGTGGAGGGTCATTG-3’
Staphylococcus epidermidis	
vraR	FP 5’-GCGCTTGCAATCTCTTGGTT-3’
	RP 5’-TAGCTGACGCTGTGCGTAAA-3’
icaA	FP 5’-CGAACCACGTGCTCTATGCT-3’
	RP 5’-TACTTCATGCCCGCCTTGAG-3’
icaD	FP 5’-GGATGTATTGTATCGTTGTGATGAT-3’
	RP 5’-GTACAAACAAACTCATCCATCCG-3’
atlE	FP 5’-TATTGGTGAGGGTCAGCGTG-3’
	RP 5’-GCTGATTATGCTGCAACGCA-3’
16srRNA	FP 5’-TCAACCGTGGAGGGTCATTG-3’
	RP 5’-AGACCAGAAAGTCGCCTTCG-3’
